# Cyclooxygenase-1 as a Potential Therapeutic Target for Seizure Suppression: Evidences from Zebrafish Pentylenetetrazole-Seizure Model

**DOI:** 10.3389/fneur.2016.00200

**Published:** 2016-11-15

**Authors:** Patrícia Gonçalves Barbalho, Benilton de Sá Carvalho, Iscia Lopes-Cendes, Claudia Vianna Maurer-Morelli

**Affiliations:** ^1^Department of Medical Genetics, School of Medical Sciences, University of Campinas, Sao Paulo, Brazil; ^2^Department of Statistics, Institute of Mathematics, Statistics and Scientific Computing, University of Campinas, Sao Paulo, Brazil

**Keywords:** zebrafish model, seizures, neuroinflammation, cyclooxygenases, anticonvulsant

## Abstract

Cyclooxygenases (COX)-1 and -2 are isoenzymes that catalyze the conversion of arachidonic acid into prostaglandins (PGs). COX-2 and PGs are rapidly increased following seizures and are known to play important roles in the neuroinflammatory process. COX-2 isoform has been predominantly explored as the most suitable target for pharmacological intervention in epilepsy studies, while COX-1 remains poorly investigated. In the present study, we evaluated the effects of selective COX-1 inhibitor or selective COX-2 inhibitor on seizure suppression in the zebrafish pentylenetetrazole (PTZ)-seizure model. Zebrafish larvae were incubated in 5 μM of SC-236 for 24 h or 2.8 μM of SC-560 for 30 min, followed by exposure to 15 mM PTZ for 60 min. Real-time quantitative PCR analysis was carried out to investigate transcription levels of *cox1* (*ptgs1*), as well as to determine *cfos* levels, used as a marker for neuronal activity. Effects of selective COX-2 or COX-1 inhibitors on locomotor activity response (velocity and distance moved) during PTZ exposure were evaluated using the Danio Vision video-tracking system. Our results showed an inducible expression of the *cox1* gene after 60 min of PTZ exposure. *Cox1* mRNA levels were upregulated compared with the control group. We found that COX-2 inhibition treatment had no effect on zebrafish PTZ-induced seizures. On the other hand, COX-1 inhibition significantly attenuated PTZ-induced increase of locomotor activity and reduced the *c-fos* mRNA expression. These findings suggest that COX-1 inhibition rather than COX-2 has positive effects on seizure suppression in the zebrafish PTZ-seizure model.

## Introduction

Seizures are conceptually defined as abnormal and hypersynchronous neuronal activity that is caused by an imbalance between excitatory and inhibitory neurotransmission (1). Thus, it is not surprising that the mechanisms of action of most antiepileptic drugs (AEDs) target neuronal mechanisms involving in the modulation of voltage-activated ion channels, inhibition of excitatory, or enhancement of inhibitory synaptic neurotransmission (2). However, approximately 30% of patients remain pharmacoresistant to conventional drug-therapy (3). Therefore, a better understanding of the molecular mechanisms involved in ictogenesis and epileptogenesis is crucial for the discovery of new drugs with anticonvulsant properties (4–6).

There is accumulated experimental evidence supporting the contribution of non-neuronal cells, astrocytes, and microglia, to the pathophysiology of epilepsy (7–12). It is known that astrocytes and microglia activation promotes the release of proinflammatory mediators, such as interleukin-1 beta (IL-1β), interleukin-6 (IL-6), tumor necrosis factor-alpha (TNF-α), cyclooxygenase (COX)-2, and prostaglandins (PGs) (13, 14). Experimental studies suggest that proinflammatory mediators can affect the physiological functions of glio-neuronal communications, thus contributing to neuronal hyperexcitability and seizure-related neuronal damage (15–17). Therefore, inflammatory mediators have been explored as alternative pharmacological targets for therapeutic intervention to treat epilepsy (11, 18, 19).

Cyclooxygenase-2 and PGs are rapidly increased following seizures and are known to play important roles in the neuroinflammatory process (19). Prostaglandin endoperoxide synthases (PTGS) or COX-1 and COX-2 are isoenzymes that catalyze the conversion of arachidonic acid into PGs (20). COX-1 and COX-2 have been traditionally classified into constitutive and inducible expression, respectively (20). Because *COX-2* is considered the inducible expressed isoform responsible for propagating the inflammatory response, several studies have been predominantly exploring the COX-2 isoform as the most suitable target for pharmacological intervention in epilepsy studies (11, 21, 22). However, the role of COX-2 inhibition on epileptogenesis and/or seizure suppression remains controversial.

Dhir et al. (23, 24) reported that pretreatment with both, selective and non-selective COX-2 inhibitors in the pentylenetetrazole (PTZ)-kindling model in rats, decreased the behavioral and biochemical parameters used as kindling score. In contrast, Claycomb et al. (25) reported that the pretreatment with rofecoxib, a selective COX-2 inhibitor, did not attenuate kindling progress in the PTZ-kindled mice. Recently, Katyal et al. (26) showed that the selective COX-2 inhibitor, etoricoxib, presented an anticonvulsant effect in the PTZ-kindled rats. In addition, treatment with nimesulide, a COX-2 selective inhibitor, prior to electrical kindling, had antiepileptogenic effects in rodents (27, 28). Furthermore, it was reported that the treatment with selective COX-2 inhibitor (SC58236) administered during the latent period did not modify epileptogenesis or chronic seizure activity after electrically induced *status epilepticus* (SE) in rats (29). On the other hand, the treatment with the SC58236 administered prior to electrically induced or during the chronic period increased seizure frequency and mortality rate (30). Proconvulsant effects of COX-2 inhibitor administration have also been reported in kainic acid-induced seizure model (31, 32). Conflicting outcomes regarding selective COX-2 inhibition have been reported in the pilocarpine-induced SE model. It has been shown that the administration of celecoxib, a selective COX-2 inhibitor, after a pilocarpine-induced SE in rats had an antiepileptogenic effect (33). In contrast, Polascheck et al. (34) show that the selective COX-2 inhibitor, parecoxib, after a pilocarpine-induced SE in rats had no antiepileptogenic effect.

Regarding COX-1 studies in animal models of seizures/epilepsy, Tanaka and colleagues reported that the selective COX-1 inhibitor slowed the development of epilepsy in electrical amygdala kindling in mouse model (35). Moreover, NSAIDs as indomethacin and aspirin, which inhibit the activity of both COX-1 and COX-2, reduced seizures in the absence epilepsy model, in the zebrafish seizure model, and in the pilocarpine-induced SE model (35–37). Nonetheless, the pharmacological inhibition of COX-1 isoform in acute and chronic epilepsy models remains poorly investigated. Therefore, in the present study we evaluated the effects of selective COX-1 inhibitor or selective COX-2 inhibitor on seizure suppression by evaluating *c-fos* mRNA expression and locomotor activity response in the zebrafish seizure model by evaluating the *c-fos* mRNA expression and locomotor activity response.

## Materials and Methods

### Animals

Wild-type adult fish were housed in 30–50 l tanks (two animals per liter) filled with non-chlorinated water cleared with mechanical and chemical filtration. Adult fish were maintained at 26 ± 2°C and in a simulated photoperiod cycle of 10 h dark/14 h light. Adult fish were fed twice a day with commercial flake fish food (Tetramin, Tetra, Blacksburg, VA, USA) and once a day with artemia; larvae were fed with paramecium and artemia twice a day. Fertilized eggs were collected after natural spawning. Embryos and larvae were housed using Petri dishes filled with water in an incubator system at the same temperature and photoperiods that were used for maintaining the adults. All experimental protocols used in this study were reviewed and approved by the Ethical Committee for Animal Research of the University of Campinas (#3098-1 and #4081-1).

### Chemicals

5-(4-Chlorophenyl)-1-(4-methoxyphenyl)-3-trifluoromethyl pyrazole (SC-560), 4-[5-(4-Chlorophenyl)-3-(trifluoromethyl)-1H-pyrazol-1-yl]-benzenesulfonamide (SC-236), and PTZ were purchased from Sigma-Aldrich (St. Louis, MO, USA).

### Pentylenetetrazole Treatment

Detailed experimental procedures have been described previously (37). Briefly, 7 days post fertilization (dpf) larvae were placed in a 24-well plate (one larva per well) containing 15 mM PTZ [seizure group (SG)] or PTZ-free water [control group (CG)] for 60 min. Following, animals were cryoanaesthetized and their heads were isolated, quickly frozen in liquid nitrogen, and stored at −80°C until further processing. A total of five samples (*n* = 5) were used for each CG and SG, and each sample was composed by pooling five larval heads.

### Pharmacological Pretreatment

Zebrafish larvae were incubated in 5 μM of SC-236 containing DMSO >0.01% or 2.8 μM of SC-560 containing >0.01% DMSO in Petri dishes for 24 h and 30 min, respectively. Those concentrations were selected based on the study of Teraoka et al. (38). After the incubation, they were exposed to 15 mM PTZ for 60 min as described above.

### Real-time Quantitative PCR

Real-time quantitative PCR (qPCR) analysis was carried out to investigate the transcript levels of *cox1* (*ptgs1*) and *cfos* (*fos*), the last one used as a marker for neuronal activity. Total RNA extraction, reverse transcription, and qPCR were performed as previously reported (37). Briefly, total RNA was extracted from each group samples (control; seizure; SC-236; vehicle (DMSO)-treated controls; and SC-560 groups) using TRIzol^®^ (Invitrogen, Carlsbad, CA, USA) according to the manufacturer’s instructions, and its concentration and quality were determined with the Epoch™ spectrophotometer (BioTek, Winooski, VT, USA) and electrophoresis using agarose gels. cDNA was generated using the High Capacity first-strand synthesis system for RT-PCR (Invitrogen, Carlsbad, CA, USA) according to the manufacturer’s instructions. Relative mRNA quantification was performed using the ABI 7500 Real-Time PCR system (Applied Biosystems, Foster City, CA, USA) with LuminoCt^®^ qPCR ReadyMix (Sigma-Aldrich, St. Louis, MO, USA) and TaqMan^®^ Gene Expression Assay (Invitrogen, Carlsbad, CA, USA). Runs were carried out in triplicate using the housekeeping gene *eef1a1l1* (Dr03432748_m1) to normalize the mRNA level of *ptgs1* (Dr03087197_m1) and *c-fos* (*fos*) (Dr03100809_g1). Data were analyzed using the SDS 7500 software (Applied Biosystems) to estimate qPCR efficiency and quantify the relative gene expression. A total of five samples (*n* = 5) were used for each group and each sample comprising a pool of five larval heads.

### Behavioral Seizure Locomotor Activity

At 7 dpf, zebrafish larvae were transferred to 96-well plate in a semi-randomized distribution (in order to have larvae distributed in lines and columns along the plate) using a micropipette in a volume of 50 μl and acclimated for 30 min to minimize any interference in the test. Following acclimation, 50 μl of aquarium water or 50 μl of a 30 mM PTZ solution (dissolved in aquarium water) was added to obtain final volume and concentration of 15 mM. After that, the locomotor activity for each larva was recorded during 30 min using an automated computerized video-tracking system DanioVision (Noldus, Wageningen, The Netherlands). A total of seven zebrafish larvae (*n* = 7) were used to compose each experimental group: (i) control, (ii) seizure, (iii) vehicle (DMSO)-treated controls, (iv) SC-236, or (v) SC-560 PTZ-treated groups. The total distance moved and the speed (velocity) for each larva from each group was quantified using the EthoVision XT locomotion tracking software (Noldus, Wageningen, The Netherlands).

### Statistical Analysis

Data are presented as mean values ± SEM. Statistical analysis was performed using the GraphPad Prism version 5.0 (GraphPad Software, San Diego, CA, USA). In all the analyses, the significance level was set at *p* ≤ 0.05. Statistical comparisons between two groups were performed using the Mann–Whitney test. Statistical comparisons between more than three groups were assessed by one-way analysis of variance (ANOVA) followed by Bonferroni’s *post hoc* test.

## Results

### Evaluation of *cox1* mRNA Expression after PTZ-Induced Seizures

**Figure 1:**
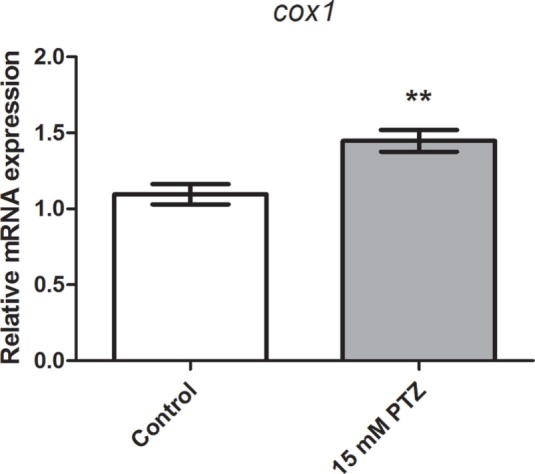
**Evaluation of *cox1* mRNA expression after PTZ-induced seizures**. Relative quantification of cyclooxygenase-1 transcript levels following 60 min of pentylenetetrazole (PTZ)-induced seizures in the zebrafish brain at 7 days post fertilization. Seizure group was exposed to 15 mM PTZ, and the control group was handled identically, but in PTZ-free water (*n* = 5 per group). Data are presented as mean ± SEM. Statistical differences between control and seizure groups were tested using the Mann–Whitney test. Two asterisks (**) indicated that *p* ≤ 0.01.

We previously reported that *cox2b* mRNA expression was upregulated after PTZ-induced seizures in 7 dpf zebrafish larva (37). To determine whether the *cox1* transcription is affected following PTZ-induced seizure, we used real-time qPCR to measure the relative quantification of the *cox1* gene. Our results showed an inducible expression of *cox1* after 60 min of PTZ exposure. *Cox1* mRNA levels were upregulated compared with the control group (*p* = 0.004; Figure 1). The mean ± SEM of control and PTZ groups were 1.1 ± 0.07 and 1.5 ± 0.07, respectively.

### Effects of Selective COX-1 or COX-2 Inhibitors on *c-fos* mRNA Expression

**Figure 2:**
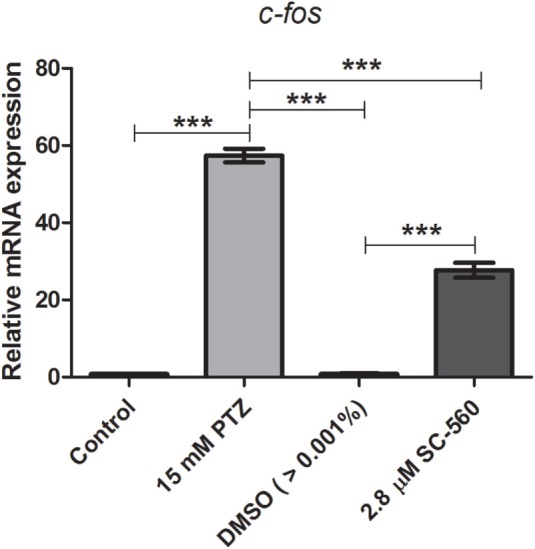
**Effect of selective COX-1 inhibitor (SC-560) on *c-fos* mRNA expression**. Seizure group (SG) was composed of animals exposed to 15 mM PTZ for 60 min. The SC-560 group (2.8 μM) was composed of animals that received SC-560 treatment for 30 min prior to PTZ exposure. Animals of the control group (CG) were handled identically but included exposure to water [no PTZ, DMSO (>0.01%), or SC-560 treatments; *n* = 5 per group]. Data are presented as mean ± SEM. One-way ANOVA followed by Bonferroni’s *post hoc* test was performed to determine statically significant differences between groups. Three asterisks (***) indicated that that *p* ≤ 0.001.

**Figure 3:**
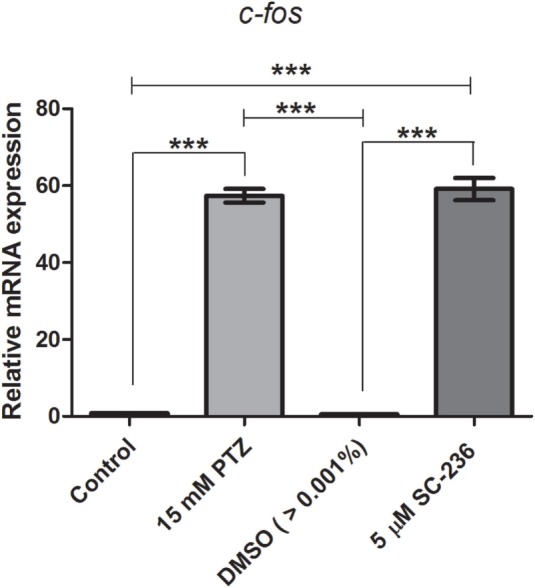
**Effect of selective COX-2 inhibitor (SC-236) on *c-fos* mRNA expression**. Relative quantification of c-fos transcript levels immediately after pentylenetetrazole (PTZ)-induced seizures in zebrafish brain at 7 days post fertilization. Seizure group (SG) was composed of animals exposed to 15 mM PTZ for 60 min. The SC-236 group (5 μM) was composed of animals that received SC-236 treatment prior to PTZ exposure. Animals in the control group (CG) were handled identically but included exposure to water [no PTZ, DMSO (>0.01%), or SC-560 treatments; *n* = 5 per group]. Data are presented as mean ± SEM. One-way ANOVA followed by Bonferroni’s *post hoc* test was performed to determine statically significant differences between groups. Three asterisks (***) indicated that that *p* ≤ 0.001.

We previously showed that the treatment with indomethacin, a non-selective COX-1 and COX-2 inhibitor, prior to PTZ-induced seizures suppresses the *c-fos* mRNA upregulation (37). Here, we sought to address the contribution of either a COX-1 or COX-2 inhibition on *c-fos* mRNA expression. Pretreatment with the selective COX-1 inhibitor, SC-560 (2.8 μM), significantly decreased the *c-fos* mRNA expression compared to PTZ group (Figure 2). The mean ± SEM obtained were (i) control group: 0.82 ± 0.08; (ii) seizure group (PTZ): 57.4 ± 1.77; (iii) vehicle (DMSO)-treated control (>0.01%) group: 0.57 ± 0.09; and (iv) SC-560 group: 27.72 ± 1.9 (Figure 2). However, our results showed that the selective COX-2 inhibitor, SC-236, treatment had no effect on *c-fos* mRNA expression (Figure 3). The mRNA levels of SC-236 group were similar with those found in the PTZ group (Figure 3). The mean ± SEM obtained were (i) control group: 0.82 ± 0.08; (ii) seizure group (PTZ): 57.4 ± 1.77; (iii) vehicle (DMSO)-treated control (>0.01%) group: 0.57 ± 0.05; and (iv) SC-236 group: 59.16 ± 2.9.

### Effects of Selective COX-1 or COX-2 Inhibitors on Locomotor Activity Response during PTZ Exposure

**Figure 4:**
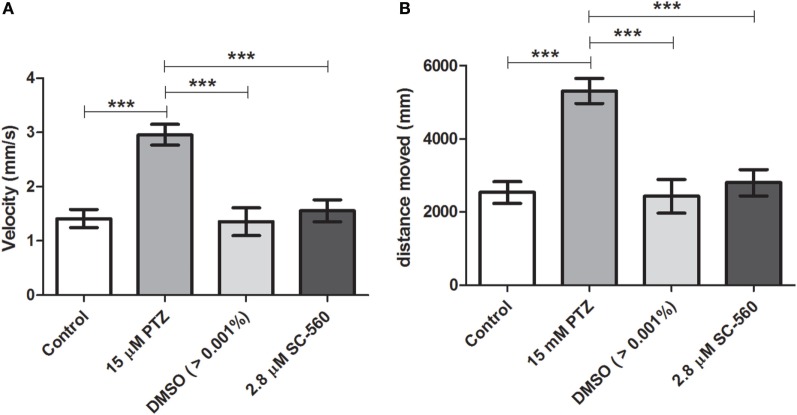
**Effect of the selective COX-1 inhibitor on locomotor activity response during PTZ exposure**. **(A)** Velocity and **(B)** distance moved. Animals were exposed to 2.8 μM of SC-560 for 30 min prior to pentylenetetrazole (15 mM) exposure. Animals in the control group (CG) were handled identically but included exposure to water [no PTZ, DMSO (>0.01%), or SC-560 treatments; *n* = 7 per group]. At 7 days post fertilization, larval zebrafish were transferred to 96-well plate and acclimated for 30 min to minimize any disturbance related to handling and transport from the Petri dishes to 96-well plate. Following acclimation, locomotor activity for each treatment group was recorded for 30 min using an automated video-tracking system Danio Vision (Noldus, Wageningen, The Netherlands). The distance moved and the velocity for each larva from each group was performed using EthoVision XT locomotion tracking software (Noldus, Wageningen, The Netherlands). Data are presented as mean ± SEM. One-way ANOVA followed by Bonferroni’s *post hoc* test was performed to determine statically significant differences between groups. Three asterisks (***) indicated that that *p* ≤ 0.001.

**Figure 5:**
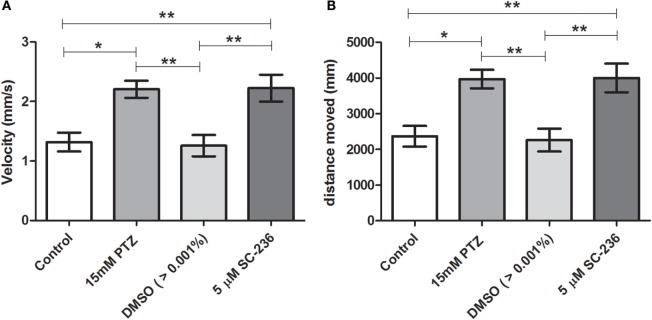
**Effect of selective COX-2 inhibitor (SC-236) on locomotor activity response during PTZ exposure**. **(A)** Velocity and **(B)** distance moved. Animals were exposed to 5 μM of SC-236 for 24 h prior to pentylenetetrazole (15 mM) exposure. Animals in the control group (CG) were handled identically but included exposure to water [no PTZ, DMSO (>0.01%), or SC-236 treatments; *n* = 7 per group]. At 7 days post fertilization, larval zebrafish were transferred to a 96-well plate and acclimated for 30 min to minimize any disturbance related to handling and transport from the Petri dishes to the plate. Following acclimation, locomotor activity for each treatment group was recorded for 30 min using an automated video-tracking system Danio Vision (Noldus, Wageningen, The Netherlands). The distance moved and the velocity for each larva from each group was performed using EthoVision XT locomotion tracking software (Noldus, Wageningen, The Netherlands). Data are presented as mean ± SEM. One-way ANOVA followed by Bonferroni’s *post hoc* test was performed to determine statically significant differences between groups. One asterisk (*) indicated that *p* ≤ 0.05; two asterisks (**) indicated that *p* ≤ 0.01.

Our initial pilot experiment revealed that after 1 h of PTZ exposure there was no difference in velocity and distance moved between PTZ and the control groups. As pointed out by Afrikanova et al. (39), the decline of the locomotor activity observed in longer periods of PTZ exposure might be due to the fact that the larvae begin to undergo seizures with more frequency and spending more time on seizure stage III (loss of posture), which, as consequence, promotes a decrease of the locomotor activity. Therefore, we choose to evaluate the locomotor activity during 30 min of PTZ exposure. Our results have shown that SC-560 treatment inhibits the PTZ-induced increase of locomotor activity (Figure 4). The velocity and distance moved of the SC-560 group were lower in comparison to the PTZ group (Figures 4A,B). For velocity, the mean ± SEM of the control, seizure (PTZ), vehicle (DMSO)-treated control (>0.01%), and SC-560 groups were, respectively, 1.4 ± 0.16, 2.96 ± 0.20, 1.35 ± 0.26, and 1.55 ± 0.20. For distance moved, the mean ± SEM of control, seizure (PTZ), vehicle (DMSO)-treated control (>0.01%), and SC-560 were, respectively, 2536 ± 297.7, 5311 ± 342.5, 2432 ± 460.6, and 2802 ± 360.7. On the other hand, SC-236 treatment did not reduce PTZ-induced increase of locomotor activity (Figures 5A,B). Velocity and distance moved of the SC-236 group were similar to those found to the PTZ group (Figures 5A,B). For velocity, the mean ± SEM of control, seizure (PTZ), vehicle (DMSO)-treated control (>0.01%), and SC-236 were, respectively, 1.3 ± 0.16, 1.26 ± 0.18, 2.22 ± 0.22, and 2.20 ± 0.14. For distance moved, the mean ± SEM of control, seizure (PTZ), vehicle (DMSO)-treated control (>0.01%), and SC-236 groups were, respectively, 2365 ± 285.6, 2258 ± 322.9, 3995 ± 403.0, and 3965 ± 257.0.

## Discussion

In the central nervous system (CNS), both COX-1 and COX-2 are constitutively expressed in neurons, astrocytes, and microglial cells, and both COXs lead to PG productions, which are important inflammatory mediators (20). Experimental evidence has shown that PGs are markedly increased following seizures and may contribute to epileptogenesis and reduction in the seizure threshold (11, 17–19). Traditionally, COX-1 and COX-2 isoforms have been considered constitutive and inducible expression, respectively (40–42). Therefore, most experimental epilepsy research reports have focused on the COX-2 isoform because of its role in the inflammatory response through PGs production (18, 19). Several studies were designed to interfere with the COX-2 enzyme without affecting the homeostatic function of COX-1, by using selective COX-2 inhibitors. However, both proconvulsant and anticonvulsant effects of COX-2 inhibition have been reported, and their role still remains controversial (11, 19, 20).

It has been reported that the therapeutic outcome of COX-2 inhibition on epileptogenesis and/or seizure suppression appears to depend on many factors including the type of inhibitor used (non-selective or selective) and the timing of the treatment administration (prior or after to seizure onset) (11, 36). Moreover, comparisons between these conflicting reports are difficult to be interpreted because they used different methods for seizure-induction, were evaluated in mature or immature brain, were assessed in different phase of epileptogenesis as well as were performed in different species (11, 36). Nonetheless, studies using non-selective COX inhibitors in the zebrafish seizure model or in the absence epilepsy rodent model as well as following pilocarpine-induced SE, showed anticonvulsant effects (35–37). Thus, these data indirectly suggest the involvement of the COX-1 isoform on seizure inhibition in these models.

Notably, experimental studies have reported neuroprotective effects of selective COX-1 inhibition and NSAIDs, with higher selectivity for COX-1, in models of neurodegenerative diseases such as Alzheimer disease and traumatic brain injury (41–49). It is noteworthy, however, that continuous NSAIDs use is associated with gastrointestinal side effects due to COX-1-derived PGs inhibition (42, 50). On the other hand, the continuous use of selective COX-2 inhibitors (coxibs) has been associated with increased risk of cardiovascular side effects (42, 50). Therefore, the benefit–risk evaluation of the administration of selective or non-selective COX inhibitors, as for many other drugs, should be considered.

Although several studies have suggested that COX-1 might have an important role in the neuroinflammation of neurodegenerative diseases (40–42), only few studies have investigated the association between COX-1 and epilepsy (35, 36). Notably, COX-1 expression has increased following kindling progression and in the SE induced by pilocarpine (35, 36). Moreover, treatment with selective COX-1 inhibitor reduced the epileptogenesis in the electrical kindling model (35). Based on this background, we sought to investigate the contribution of each COX isoforms on seizure suppression in the zebrafish PTZ-seizure model.

Although it is considered as an acute seizure model, the zebrafish is a powerful model in neuroscience research since it has many advantages when performing imaging studies, genetic manipulation, and modeling different disease mechanisms. In addition, it is a convenient model for drug screening, making it suitable for translational studies in epilepsy (51–53).

The zebrafish has homologs for both COX-1 and COX-2 human genes named *cox1* (*ptgs1*) and *cox2* (*ptgs2*), respectively (54). Furthermore, the zebrafish PTZ-seizure model has been widely used as an *in vivo* high-throughput screening method for antiepileptic drug discovery (55–57). Screening of new compounds with anticonvulsants’ properties is performed by evaluating the inhibition of the seizure-like behavior (i.e., by assessing the locomotor activity), as well as by the suppression of the increased neuronal activity, through the *c-fos* quantification in the brain and/or by the inhibition of epileptiform electrographic activity (39, 55, 58).

In this study, we showed that the *cox1* mRNA expression was increased after PTZ-induced seizure (Figure 1). In addition, we found that the selective COX-1 inhibitor, SC-560, significantly decreased the *c-fos* mRNA expression (Figure 2) and inhibited the PTZ-induced increase of locomotor activity (Figure 4). Taking all together, these findings suggest that COX-1 inhibition has an important role on seizure suppression in the zebrafish seizure model, whereas the selective COX-2 inhibitor (SC-236) had no effect, producing any changes in the *c-fos* mRNA expression (Figure 3) and locomotor activity (Figure 5).

Our findings suggest that inhibition of the COX-1 isoform has an important role on seizure suppression in the zebrafish PTZ-seizure model. Together, with the accumulating knowledge arising from other animal models of epilepsy and given the emerging importance of the zebrafish as a model for epilepsy investigations and drug screening, we believe these findings may contribute with new avenues of investigations toward seizure suppression.

## Author Contributions

PB performed the experiments, participated in the design of the study, analyzed the data, and wrote the paper. BC analyzed the statistical data. IL-C contributed with the laboratory support and revised the manuscript. CM-M conceived the study, participated in its design, coordination, and revised the manuscript.

## Conflict of Interest Statement

The authors declare that the research was conducted in the absence of any commercial or financial relationships that could be construed as a potential conflict of interest.
